# Formation of carbyne-like materials during low temperature pyrolysis of lignocellulosic biomass: A natural resource of linear sp carbons

**DOI:** 10.1038/s41598-017-17240-1

**Published:** 2017-12-04

**Authors:** Rita Khanna, Muhammad Ikram-Ul-Haq, Aditya Rawal, Ravindra Rajarao, Veena Sahajwalla, Romina Cayumil, Partha S. Mukherjee

**Affiliations:** 1 Centre for Sustainable Materials Research and Technology, School of Materials Science and Engineering, The University of New South Wales, NSW 2052 Sydney, Australia; 2 Nuclear Magnetic Resonance Facility, Mark Weinwright Analytical Centre, The University of New South Wales, NSW 2052 Sydney, Australia; 3 Department of Metallurgical Engineering, Faculty of Engineering, Andrés Bello University, Santiago, Chile; 4 Institute of Minerals and Materials Technology, Advanced Materials Technology Department, Bhubaneshwar, Orissa, 751013 India

## Abstract

The exploration, understanding and potential applications of ‘Carbyne’, the one-dimensional sp allotrope of carbon, have been severely limited due to its extreme reactivity and a tendency for highly exothermic cross-linking. Due to ill-defined materials, limited characterization and a lack of compelling definitive evidence, even the existence of linear carbons has been questioned. We report a first-ever investigation on the formation of carbyne-like materials during low temperature pyrolysis of biobased lignin, a natural bioresource. The presence of carbyne was confirmed by detecting acetylenic –C≡C– bonds in lignin chars using NMR, Raman and FTIR spectroscopies. The crystallographic structure of this phase was determined as hexagonal: *a* = 6.052 Å, *c* = 6.96 Å from x-ray diffraction results. HRSEM images on lignin chars showed that the carbyne phase was present as nanoscale flakes/fibers (~10 nm thick) dispersed in an organic matrix and showed no sign of overlapping or physical contact. These nanostructures did not show any tendency towards cross-linking, but preferred to branch out instead. Overcoming key issues/challenges associated with their formation and stability, this study presents a novel approach for producing a stable condensed phase of sp-bonded linear carbons from a low-cost, naturally abundant, and renewable bioresource.

## Introduction

Remarkable developments based on carbon’s ability to bind to itself in a variety of hybridized states (sp^3^, sp^2^, sp) have seen the emergence of synthetic carbons with exceptional characteristics, e.g., fullerenes, carbon nanotubes, graphene, C_8_, lonsdaleite etc.^1–3^. Unique mechanical, optical and electronic properties of these materials have led to significant scientific advances and technological breakthroughs^4,5^. The sp hybridized allotrope of carbon, ‘Carbyne’ – also called linear acetylenic carbon– an infinite linear chain of carbon atoms with alternating single and triple bonds, has been the subject of intense research^6–8^. Although detected in trace amounts in meteorite craters, interstellar dust, carbonaceous chondrites etc.^9^, the carbyne phase has remained elusive, little known and controversial despite extensive investigations. Pure carbynes are unstable in the condensed phase due to their extreme reactivity^10,11^. During synthesis, linear carbon chains have been artificially stabilized by using heavy end-capping and/or pendant side groups, matrix isolation of chains at extremely low temperatures to prevent cross-linking reactions and to protect from decomposition in a reactive environment, the use of solution dispersion, ultrahigh vacuum etc.^12–14^. Shi *et al*. have produced long acetylenic carbon chains encapsulated within thin double-walled carbon nanotubes by physically preventing these chains from coming together^15^. Computed to be stronger than any known material including diamond, the tensile stiffness of carbyne has been theoretically estimated to be twice that of graphene and carbon nanotubes^16^. With outstanding thermo-stability, non-linear optical behaviour and biocompatibility, linear carbon chains have been regarded as a potential minimal nanowire, the thinnest connection possible for an ultimate device for applications in nanoelectronics, transport channels, molecular and superionic devices^17,18^.

In 1978, Whitaker had reported on detecting naturally occurring forms of carbyne found dispersed in natural graphite sourced from mines across the globe; these had been in existence for at least sixty million years^19^. Flakes of natural carbyne have also been reported in diamond mines of china^20^. Could the carbyne phase form naturally in biomass/organic matter, a well-known source of carbonaceous materials such as amorphous carbon, coals, cokes, graphite, fossil fuels etc.? We carried out low temperature (200–800 °C) pyrolysis investigations on biobased lignin, a coproduct of bioethanol production from sugarcane bagasse^21^. This carbon-rich biomaterial with inherent mineral impurities was chosen to mimic natural plant/organic matter as closely as possible. In this article, we present a first-ever report on the formation of carbyne like materials (identified by the presence of acetylenic –C≡C– bonds) in lignin chars after heat treatment at 400 °C for 30 minutes in argon atmosphere. The carbyne phase made its first appearance at 375 °C, developed significantly at 400 °C, and was no longer present at 600 °C. A systematic characterization of these lignin chars was carried out using a variety of advanced analytical techniques such as NMR, Raman & FTIR spectroscopies, XRD, and HRSEM/EDS. Detailed results from these investigations are presented next.

## Results and Discussion

**Figure 1:**
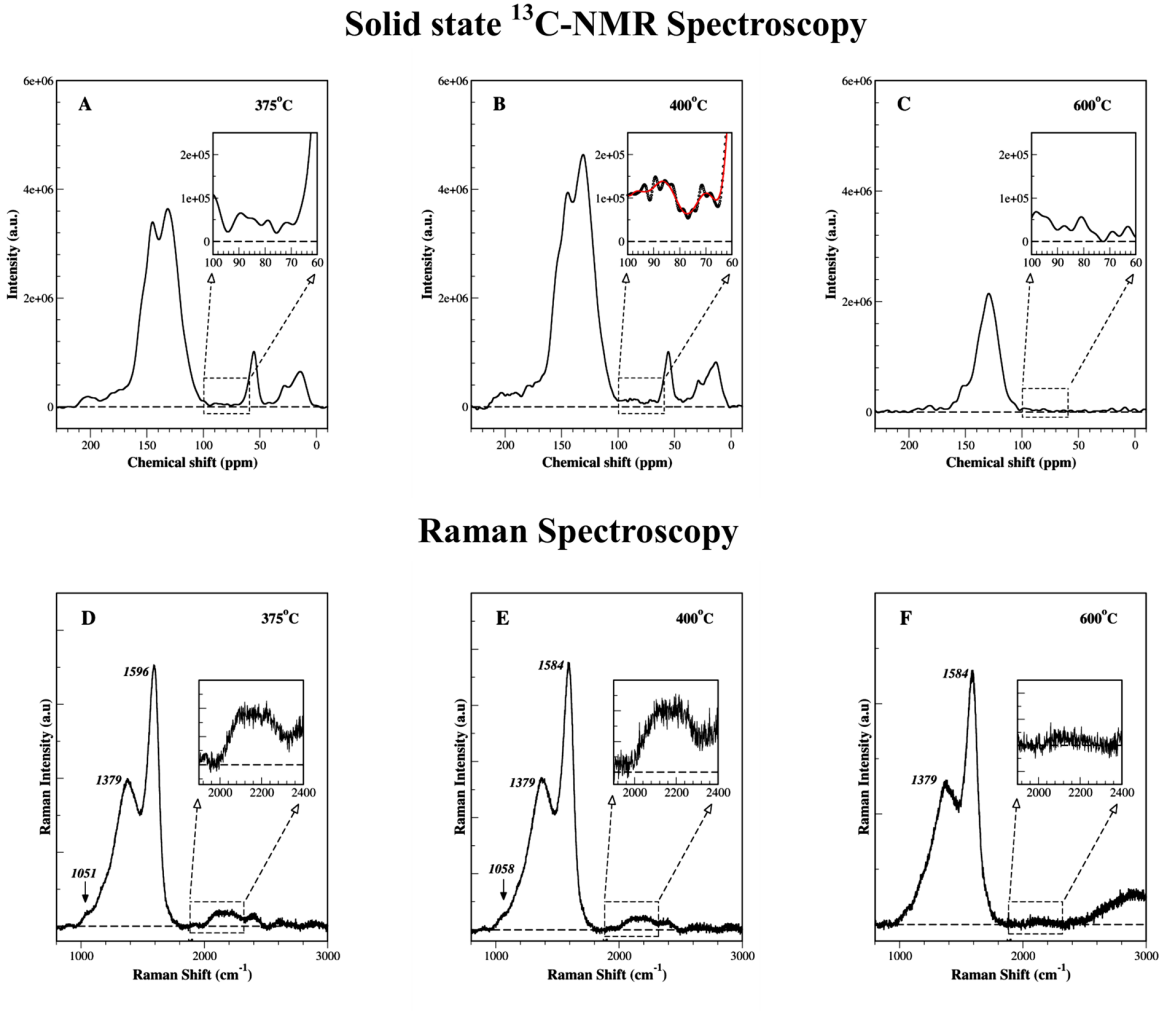
Spectroscopic analysis of lignin chars. (**A–C**) NMR and (**D–F**) Raman spectroscopy results on 375 °C, 400 °C and 600 °C lignin chars. Insets in these figures represent spectral regions specific to the location of −C≡C− alkyne bonds.

Figure 1(A–C) shows the non-protonated solid state ^13^C NMR spectra of lignin chars after heat treatments at 375 °C, 400 °C and 600 °C for 30 minutes; the zero baseline was represented by a horizontal dashed line in all the spectra. Using dipolar dephasing of ^13^C signals, the contribution from the protonated, non-mobile carbon species (carbons bonded with hydrogen) was suppressed by turning off the ^1^H decoupling for short periods (40 µs)^22^ (Fig. S1a). The chemical shift region between 100–60 ppm was highlighted as insets in these figures to locate acetylenic –C≡C– species (typical range: 95–65 ppm). The NMR signals from the 400 °C char showed two broad peaks ~88 and 69 ppm; with a signal to noise ratio of 4.25, these represent real peaks significantly above the background noise. While a small signal could be observed for the 375 °C char, the NMR signal for the 600 °C char could not be distinguished from noise in this spectral region. As the interference from oxygen bonded carbons can be serious issue in natural organic matter, their role was carefully examined in this study. Chemical shift anisotropy (CSA) recoupling studies were carried out to identify contribution, if any, from the alkyl C-O species in this region; their presence was not detected (Fig. S1b)^23^. Observed NMR results unambiguously identified the presence of carbon-carbon triple bonds in 400 °C chars. The NMR spectra of raw lignin powder has also been provided in Fig. S1c for sake of comparison.

Raman spectroscopy is a key technique used for investigating bond structure in carbonaceous materials. Raman bands in the spectral region 2000 to 2300 cm^−1^ are specifically associated with −C≡C− bonds and can be used for an unambiguous identification of the carbyne phase. Common carbon polymorphs such as amorphous carbon, graphite, diamond, fullerene etc. do not have vibrational modes in this region^24^. Figure 1(D–F) shows the Raman spectra for lignin chars after heat treatments at 375 °C, 400 °C and 600 °C for 30 minutes. These spectra were measured using 514 nm (5 mW) argon ion laser, and represent an accumulation of 4 spectra, recorded for 30 seconds in the wavenumber range 100–4000 cm^−1^. While all three chars showed the well-known G and D bands, the Raman spectra for both 375 °C and 400 °C chars also showed a weak broad band in the region 2000 to 2300 cm^−1^. A small peak was observed at ~1050 cm^−1^ as well for these two chars indicating the presence of C-H bonds. In a good agreement with NMR results, the Raman spectra for 600 °C chars did not show any signal in this spectral region, thereby indicating the absence of –C≡C– bonds in these chars. Both NMR and Raman results for 600 °C chars point to the likely disintegration/transformation of the acetylenic group into to aromatic structures, amorphous carbons or polycyclic aromatic hydrocarbons (PAHs)^25^.

**Figure 2:**
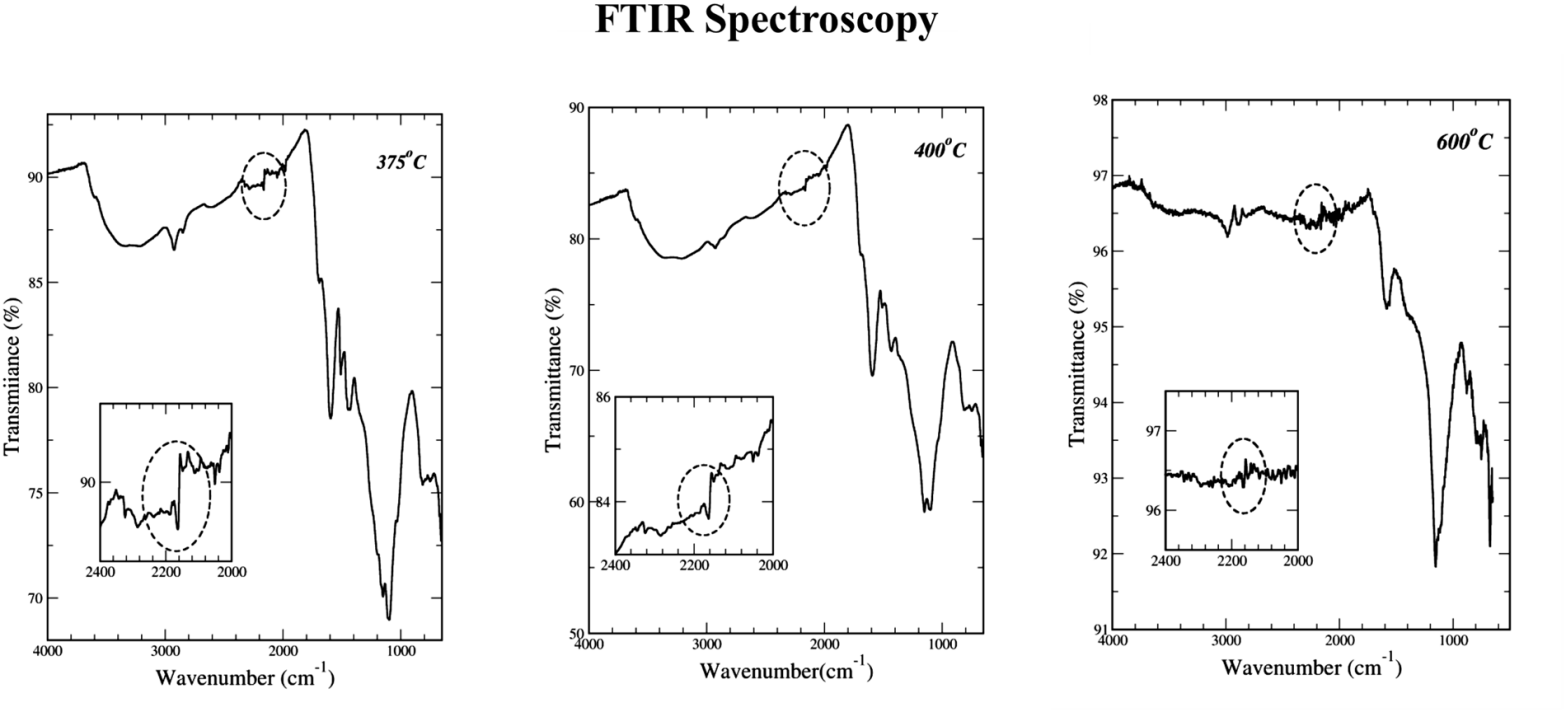
FTIR spectroscopy results for lignin chars for a range of temperatures and associated changes in the bonds present, especially the C≡C stretching vibration bond (see insets).

Detailed FTIR results on 375 °C, 400 °C and 600 °C chars are presented in Fig. 2. The spectral region between 2000 to 2400 cm^−1^ was highlighted in insets for a clear identification for the asymmetric C≡C stretching IR vibration bond. The absorption by the symmetric C≡C bond is forbidden in infrared spectroscopy due to its symmetry. These were observed as a weak but sharp discontinuity at 2160 cm^−1^ for 375 °C and 400 °C chars; the corresponding signal for the 600 °C char could not be distinguished from noise. The spectra also indicated the presence of other bonds typically present in natural organic matter, e.g., alkoxy C-O (1050–1150 cm^−1^), C=O stretch (1640–1810 cm^−1^), sp^3^ C-H stretch (2850–3000 cm^−1^), aromatic sp^2^ C-H stretch (3300 cm^−1^)^26^. With absorption frequencies for –C≡C– bonds reported to occur between 2100 and 2250 cm^−1^, the FTIR results were consistent with results from NMR and Raman spectroscopy. All three analytical techniques indicate the presence of the acetylenic carbyne phase in 375 °C and 400 °C chars, and its absence in 600 °C chars.

**Figure 3:**
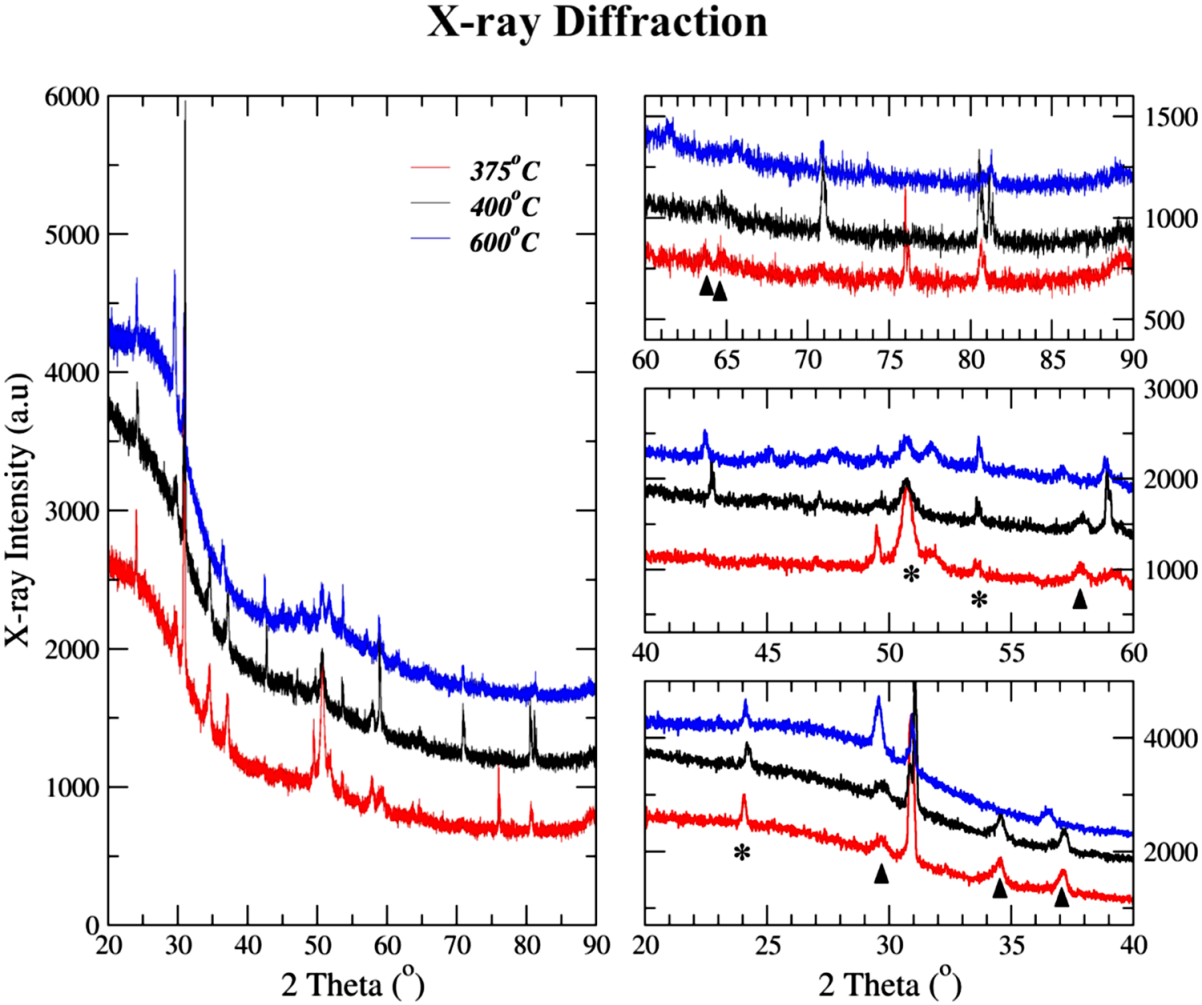
X-ray diffraction results on lignin chars. XRD plots on the right represent the expanded spectra for facilitating peak location and identification.

Figure 3 shows detailed x-ray diffraction results on lignin chars using Co K_α_ radiation; high-resolution optics was used to minimize fluorescence and scatter at low angles. The diffraction pattern was a complex mixture of peaks in a range of heights, widths and profiles, and had contributions from the carbyne phase, organic matter as well as other carbonaceous structures. Following criteria was used to identify XRD peaks for the carbyne phase: (a) all relevant peaks should have similar peak shapes and profile clearly distinct from other peaks, and (b) these peaks must only be present in 375 °C and 400 °C chars and be absent in 600 °C chars (Section S2). Additional details on indexing of sharp peaks present in 600 °C chars and their phase identification have been provided in Section S2.

Six diffraction peaks meeting these criteria were identified and marked with the symbol ‘▲’ in the expanded diffraction spectra in Fig. 3. All six carbyne peaks were indexed to a hexagonal phase with a high level of accuracy (Table 1: *a* = 6.052 Å, *c* = 6.96 Å). According to the ‘kinked chain’ model of carbyne structure^27^, these parameters indicate polyyne chains with six contiguous carbon atoms (*n* = 6) and a kink angle *α*_*p*_ = 62.43° (Polyyne range: 60° to 65°).

**Table 1:** Indexing and structural characterization details for the carbyne phase.

*Hexagonal Structure: a* = *6*.*052* *Å, c* = *6*.*96* *Å*					
S. No.	2 Theta (°)	h k l	d_o_ (Å)	d_c_ (Å)	d_o_- d_c_ (Å)
1.	29.74	002	3.4856	3.48	0.0056
2.	34.50	110	3.0164	3.027	−0.0106
3.	37.19	111	2.8052	2.7758	0.0294
4.	57.95	113	1.8465	1.8414	0.0051
5.	63.8	213	1.6958	1.6950	0.0008
6.	64.8	203	1.6694	1.6514	0.0180

**Figure 4:**
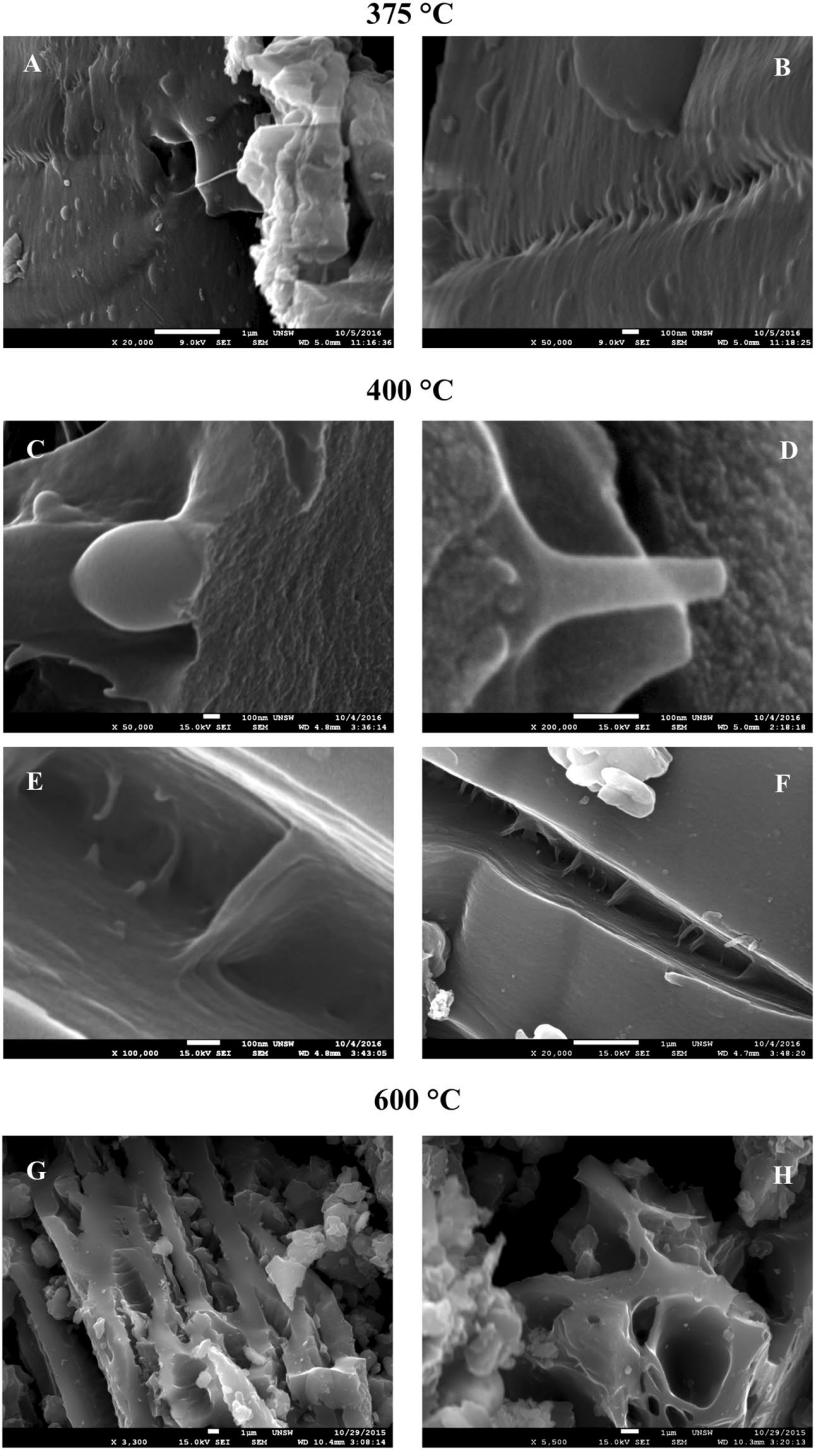
HRSEM images of lignin chars: (**A,B**) 375 °C, (**C–F**) 400 °C and (**G,H**): 600 °C, showing nanoscale features associated with the nucleation, growth and decay of the carbyne phase.

Figure 4(A–H) presents HRSEM images for three lignin chars. Figure 4(A,B) for the 375 °C char shows the emergence of numerous small (~50–200 nm) structures in the form of stretched bubbles/blisters across the surface. These formations had grown significantly at 400 °C (Fig. 4(C–F)), and were present as spheroids, elongated foils or as 20–100 nm long tubular shapes in a range of sizes/shapes. Figure 4E shows several long tubular structures in different stages of growth emerging from the inside surfaces of a cavity. Figure 4F shows some of these linking the two sides of cavity as interconnects, that may bring the two surfaces closer and seal the cavity. Several such cavities were found dispersed throughout the organic matrix (Fig. S3a). The outer rim of all these structural forms showed a whitish nanofiber outline. Several nanofiber bundles were also observed along the outer edges of the cavity.

**Figure 5:**
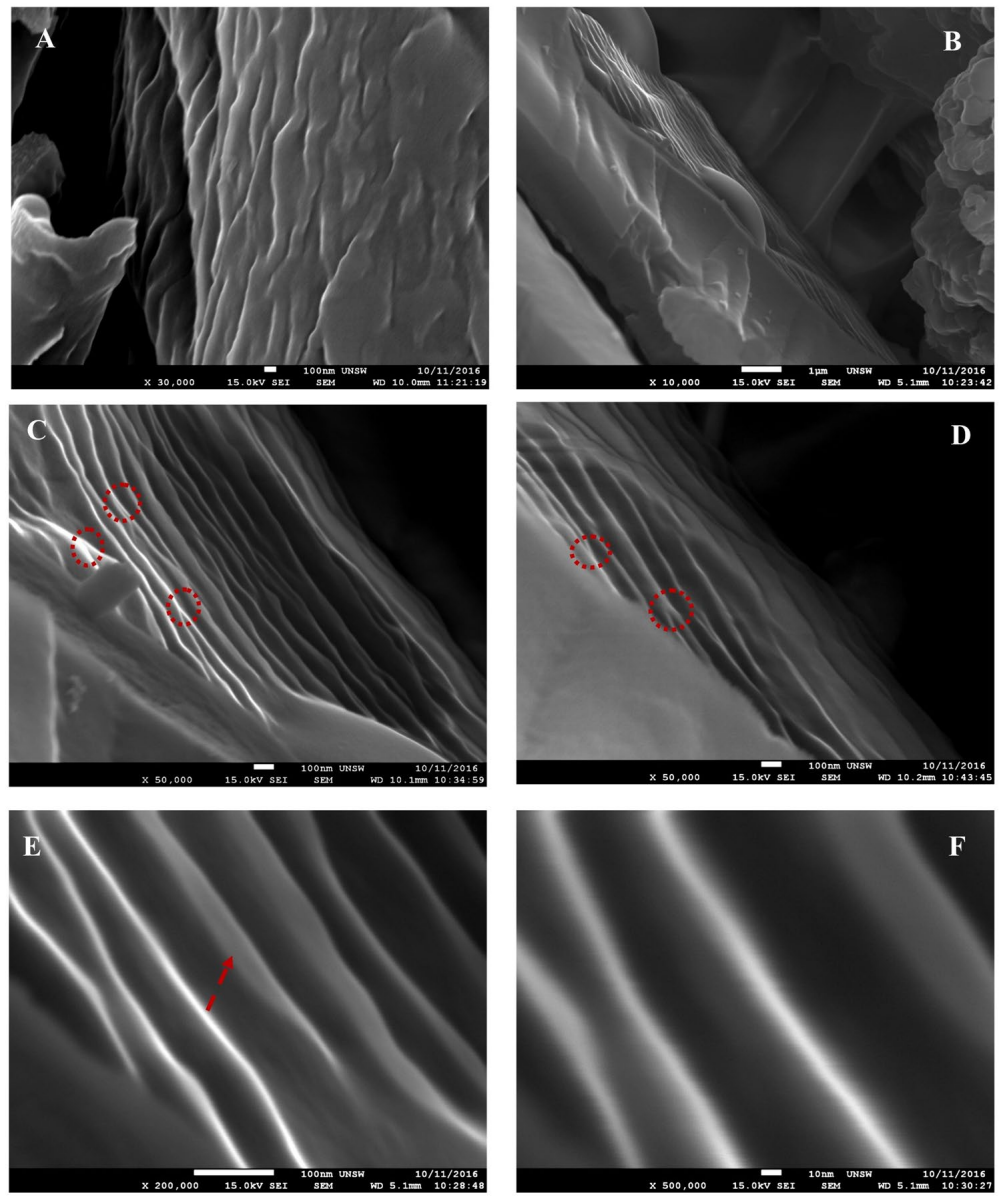
Nanoscale manifestations of linear carbons: (**A,B**) Two distinct orientations of nanofibers; (**C,D**) Branching of nanofibers at several places; and (**E,F**): Physical dimensions of nanofibers/foils and their dispersion in organic matrix.

Figure 4(G,H) for 600 °C chars showed a variety of 3D micro-structures indicating structural evolution of carbons at higher temperatures. Various nanoscale manifestations of the carbyne phase, observed in 375–400 °C chars, were completely absent in 600 °C chars. The HRSEM results were in excellent agreement with other analytical techniques regarding the nucleation/growth, thermal stability regime and subsequent decay of the carbyne phase in lignin chars. Additional details on the composition of chars and the role of cellulose/ash impurities have been provided in the section S3. Figure 5(A–H) takes a closer look at various nanofiber bundles observed in 400 °C char. Figure 5A shows a cluster of nanofibers with zagged edges in a range of lengths (>100 nm), oriented along a given direction; Fig. 5B shows nearly parallel bundles of 20 or more nanofibers several hundred nanometers long. There was no evidence for cross-linking or overlapping between various nanofibers but a clear tendency for branching out/bifurcation (Fig. 5C,D). Figure 5E,F shows several nanofibers/flakes (~10 nm thick) clearly separated from each other by 30–100 nm thick organic matter (seen as dark regions).

In this study, the carbyne phase was detected in 375 °C and 400 °C biochars; this temperature regime also marks the completion of thermal decay of cellulose (<380 °C), and the onset of lignin degradation^28^. These transformations are usually accompanied by the release of several hydrogen-bearing gases, e.g. H_2_, CH_4_, C_2_H_4_, C_2_H_6_ (dominant gas: CH_4_)^29^. The ^13^C NMR spectra of these chars (Fig. 1(A,B)) showed strong peaks in the chemical shift region between 170–110 ppm, representing a significant presence of aromatic as well as quaternary carbons. Although reaction pathways are not yet fully understood, the mechanism of carbyne formation in biochars could occur through dehydrogenation of aromatic networks and/or clusters, nucleation of a new phase and subsequent structural growth. Tabata *et al*.^30^ had proposed a similar pathway for polyyne formation during laser ablation of carbonaceous targets. Further studies on number of biochars including thermodynamics and thermochemical investigations are currently underway towards elucidating reaction energetics and kinetics.

## Conclusions

This study presents a novel, alternate route for preparing naturally stable carbyne-like materials through structural transformations of biobased lignin along with in-depth characterization. Two key factors limiting the stability of carbyne phase were overcome naturally in lignin chars: (a) preference for branching instead of cross-linking/overlapping, and (b) a natural separation between nanostructures through dispersion in an organic matrix. While further research will be required for enhancing the concentration, isolation and extraction of carbyne from the organic matrix, identifying key reaction pathways and associated energetics, our research has paved the way for producing this elusive carbon phase from an abundant, renewable bio-resource towards exploitation of its outstanding characteristics.

## Methods

### Materials and thermal treatments

The starting lignocellulosic material in these investigations was a coproduct of bio-conversion of inedible sugarcane bagasse into bio-ethanol and lignin residue^31^. Detailed initial characterization of the residual lignin was as follows: Ultimate analysis (wt. %)− C (54.0), N (1.44), S (0.4), H (5.1), O (39.06); Proximate analysis (wt. %)− fixed C (26.3), ash (4.0), volatiles (64.6), moisture (5.1); Ash analysis (wt. %)− SiO_2_ (69.73), SO_3_(12.92), CaO (10.09), Fe_2_O_3_(1.41), Al_2_O_3_ (0.86), K_2_O (0.39), P_2_O_5_(0.37), Cr_2_O_3_ (0.37), Na_2_O(0.3), MgO(0.22) and TiO_2_(0.22). The relative fractions of cellulose, lignin and hemi-cellulose in the residual lignin were determined to be 42%, 35% and 23% respectively.

Lignin powders (<50 µm) were heat treated in the temperature range 200–600 °C under argon atmosphere for times up to 60 minutes. Using an especially designed horizontal resistance furnace, the specimens were inserted directly into the preheated furnace thereby eliminating the initial period of gradual heating. The specimens were heat treated isothermally at the preset temperature for pre-defined periods of time and then quenched rapidly. This procedure is significantly different from typical pyrolysis/thermal treatments of biomass that generally use continuous heating (e.g., TGA, DSC etc.) and heating rates of 10–60 °C/min. Further details of the furnace operation, its thermal profile, and treatment procedure, and key distinctions with other biomass pyrolysis techniques are given elsewhere^21^. As both hemicellulose and cellulose are expected to have degraded and decayed at temperatures ≤380 °C^28^; various chars recovered after heat treatments in the study (≥375 °C) are expected to be predominantly lignin based. These lignin chars were characterized using a variety of analytical techniques detailed below. The spectroscopic results on these chars had remained unchanged after two years of shelf life indicating the stability of these carbyne-like materials under ambient conditions.

### Characterization of lignin chars

The ^13^C NMR experiments were carried out on Bruker AVANCE III 300 spectrometer with a 7 Tesla superconducting magnet operating at frequencies of 75 MHz for the ^13^C nuclei. The chars were ground finely and packed into 4 mm zirconia rotors with Kel-F caps. The quantitative ^13^C Direct Polarization with Magic Angle Spinning (^13^C DPMAS) NMR spectra of the material was acquired at 12 kHz MAS with a ^13^C 90° pulse length of 4 μs, 80 kHz ^1^H SPINAL64 decoupling, and a Hahn-echo before signal detection to eliminate baseline distortion. Renishaw Invia Raman spectrometer equipped with argon ion laser (514 nm) and a microscope with 50X objective was used to record the Raman spectra. The power of the laser beam was kept below 5 mW to avoid thermal degradation of samples during measurement.

Fourier-transform infrared (FTIR) spectra were obtained in the attenuated total reflectance (ATR) mode using a Perkin Elmer Spectrum 100 spectrometer. Powdered chars were mixed with KBr in the mass ratio 1:100, and were scanned in the wave number range of 4000–650 cm^−1^ (spectral resolution of 4 cm^−1^) to analyze the functional groups present in chars^26^. X-ray diffraction data on various chars was collected using PANalytical Xpert Multipurpose X-ray Diffraction System with Co Kα radiation (45KV, 40 mA) in the angular range 10–90°, a step size of 0.025° and time per step of 30 seconds. High resolution Scanning electron microscopic investigations were carried out on JEOL 7001F FE-SEM with sub-micron resolution; the specimens were platinum coated prior to microscopy. Energy dispersive spectroscopy was carried out on Hitachi S3400 SEMs (magnification: 20 X to 20000 X) for microscopic and elemental analysis.

### Data and materials availability

All data necessary to evaluate the conclusions in this report are present in the paper and in Supplementary information. Additional data, if required, will be made available by the authors upon request.

## Electronic supplementary material

Supplementary Information
