# Supplementary material for: Formation of carbyne-like materials during low temperature pyrolysis of lignocellulosic biomass: A natural resource of linear sp carbons
Source: Sci Rep. 2017 Dec 4;7:16832. doi: 10.1038/s41598-017-17240-1 (PMC5715089; doi:10.1038/s41598-017-17240-1)
Supplement: Supplementary file 1 — Supplementary Information [file 41598_2017_17240_MOESM1_ESM.pdf]

Supplementary Materials

Formation of carbyne-like materials during low temperature pyrolysis of lignocellulosic biomass: A natural resource of linear sp carbons

Rita Khanna,[1,*] Muhammad Ikram-Ul-Haq,[1] Aditya Rawal,[2] Ravindra Rajarao,[1] Veena Sahajwalla[1], Romina Cayumil,[1, 3] Partha S. Mukherjee[4]

[1]Centre for Sustainable Materials Research and Technology, School of Materials Science and Engineering, The University of New South Wales, NSW 2052, Sydney, Australia

[2]Nuclear Magnetic Resonance Facility, Mark Weinwright Analytical Centre, The University of New South Wales, NSW 2052, Sydney, Australia

[3]Department of Metallurgical Engineering, Faculty of Engineering, Andrés Bello University, Santiago, Chile.

[4]Institute of Minerals and Materials Technology, Advanced Materials Technology Department, Bhubaneshwar, Orissa, India-751013

***Corresponding author:**

A/Prof Rita Khanna

Email: ritakhanna@unsw.edu.au

Ph.: 61-2-9385 5589

This file includes:

S1: NMR Spectroscopic analysis

S2: X-ray diffraction analysis

S3: Composition, spatial distribution and the role of impurities

References (1-2)

Supplementary Section 1: NMR Spectroscopic analysis

NMR spectroscopy of organic materials: CP/MAS/TOSS ^{13}C NMR spectra [Magic Angle Spinning (MAS); ^{13}C Cross Polarization (CP) nuclear magnetic resonance (NMR) spectra with Total Suppression of Side Bands(TOSS)] of lignin chars was acquired in this study at 12 kHz MAS with a ^{13}C 90° pulse length of 4 μs, 80 kHz ^{1}H SPINAL64 decoupling, and a Hahn-echo before signal detection to eliminate baseline distortion. NMR spectroscopy can provide comprehensive structural information especially for insoluble natural organic matter. Conventionally, the spectra of all the carbon species are acquired by turning on ^{1}H decoupling during the acquisition period. However, turning off the ^{1}H decoupling for a short period of 40 μs (i.e., gated decoupling) suppresses the signal of the protonated, non- mobile carbon species, due to dipolar dephasing of the ^{13}C signal, and yields the signal for only the non-protonated or methyl carbon species. This step can identify and suppress signals from hydrogen bonded carbon atoms.

The spectra for lignin chars after heat treatment for 30 minutes at 350 °C and 400 °C are shown in Fig. S1a. The spectra in red, acquired without gated ^{1}H decoupling, represents the spectra of all carbon species, whereas the spectra in black, acquired after 40 μs of gated ^{1}H decoupling, represents the signals from non-protonated carbon species only. The baseline is represented by the horizontal dashed line in these spectra. The signals from the –C≡C– species (the *sp* hybridized alkyne carbons) are expected to be found in the chemical shift region between 100-60 ppm (see insets). No signal was observed in this region for 350 °C char after suppressing the signal from C-H species thereby indicating the absence of –C≡C– species at this temperature. However, a significant signal for the −C≡C− species was observed in the non-protonated carbon species spectra in the black curve for the 400 °C char. The signal to noise

ratio in this region was measured to be 4.25, thereby indicating the presence of a ‘real peak’ clearly above the noise level.

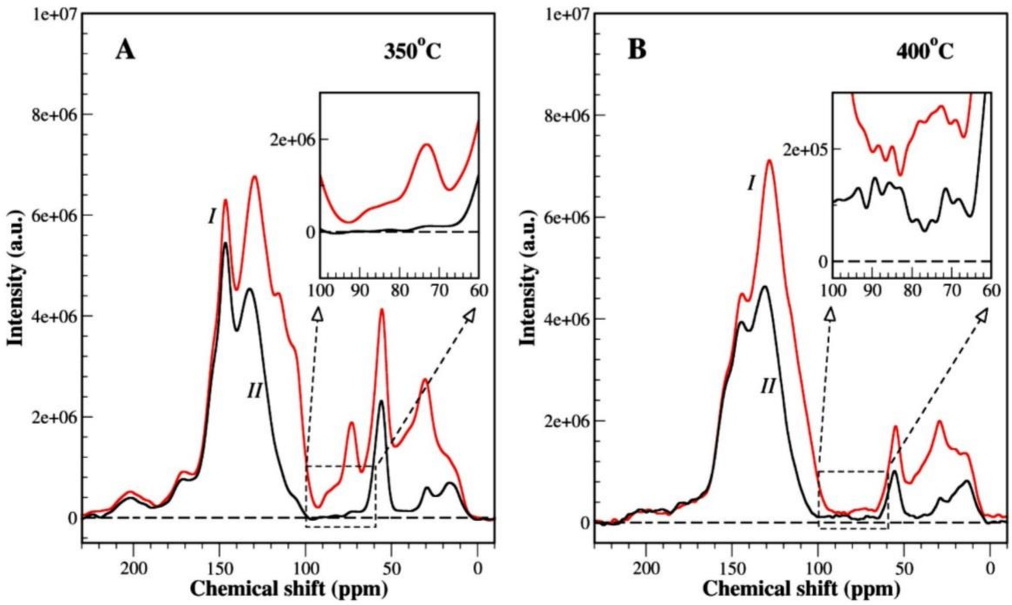

Figure S1a| ^{13}C solid state MAS NMR of lignin chars after heat treatment for 30 minutes at (A) 350°C and (B) 400 °C. The spectra in ‘red’ and ‘black’ respectively represent signals from all carbon species and only non-protonated carbons. Insets in these figures highlight the region between 100 ppm- 60ppm for locating the presence, if any, of the –C≡C– species.

The 100-60 ppm region can however have contributions from the sp^3 hybridized OCH or alkyl O-C-O carbons species, e.g., from residual sugar species; aromatic resonances down to 86 and to 95 ppm have been detected in 1,3,5-trimethyl benzene in lignins and tannins [1]. To overcome this issue, we used ‘Chemical shift anisotropy (CSA)’ to identify the local bonding symmetry of ^{13}C sites. Due to nearly symmetric tetrahedral bonding arrangement, the ^{13}C CSAs for sp^3 hybridized carbon sites ($\Delta\sigma_{aliph}$ < 70 ppm) are much smaller than the corresponding CSAs for planar sp^2 ($\Delta\sigma_{arom}$ = 120-200 ppm) and linear sp hybridized sites ($\Delta\sigma$ = 200-400

ppm). The alkyl O-C-O has small CSAs in the range 30-55 ppm. These large differences are used for an efficient separation of different types of resonances. Aromatic carbon signals with large CSAs were selectively suppressed using dephasing by the CSA and recoupled by an odd number of 180° pulses. In Fig. S1b, we show the NMR spectra for 400 °C char acquired after a 40 μs gated decoupling to select the non-protonated carbon species and 47 μs recoupling to suppress the signal from sp2/sp carbons. A complete disappearance of the signal removes the possibility of alkyl C-O species and provides further confirmation for the presence of −C≡C− in this char specimen.

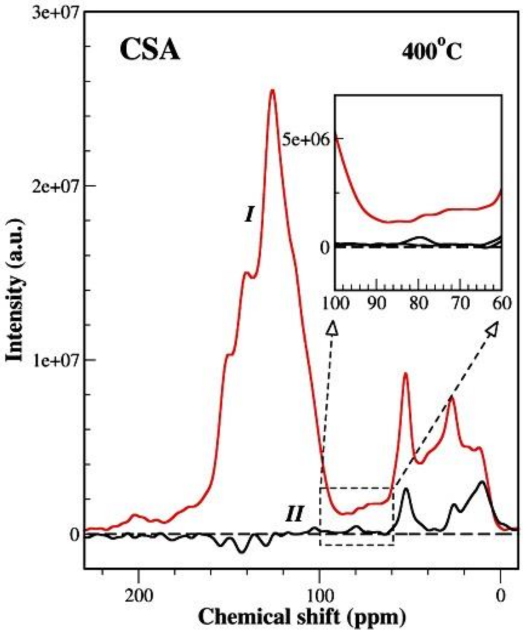

Figure S1b| ^{13}C solid state MAS NMR of lignin chars after heat treatment for 30 minutes at 400 °C. The ^{13}C NMR spectrum in ‘red’ and ‘black’ respectively represent the full spectrum and the one after 47 μs CSA recoupling.

Fig. S1c shows the NMR spectra of raw lignin powder, which contained contributions from three main constituents: cellulose (42%), lignin (35%), and hemicellulose (23%). The presence (or absence) of –C≡C– species could not be ascertained due to large contributions from several functional groups in the chemical shift region between 100-60 ppm. It is important to note that the NMR signal for the –C≡C– species was clearly absent for the 350 °C chars, and was unambiguously present in 400°C chars. The initial presence (or absence) of the –C≡C– species in the starting material may therefore not be an essential requisite for the formation of carbyne phase at higher temperatures.

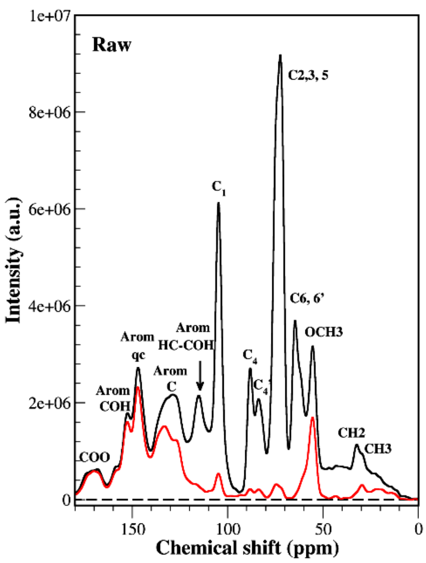

Figure S1c| ^{13}C solid state MAS NMR of raw lignin powders.

Supplementary Section 2: X-ray diffraction analysis

Peak identification and phase characterization

We had previously reported detailed X-ray diffraction investigations using Cu K_α radiation on lignin chars heat treated in the temperature range 200-800°C [21]. The diffraction pattern for the 400°C char had contributions from three distinct phases. The first set included lignin based fibers labelled as Phase 'B' that were present in 200°C as well as 400°C chars; but this phase was no longer present at 600°C. The second set included additional peaks, that made their initial appearance at 400 °C and were labelled as the phase 'C'. The XRD peaks for the 'C' phase increased in intensity at 600°C, increasing further upon heating to 800 °C. The third set included peaks that did not follow any such well-defined pattern. The peaks for the carbyne phase were not identified unambiguously due to their relatively low intensities.

Spectroscopic investigations (NMR, Raman and FTIR) on 400 °C lignin chars had indicated the presence of small amounts of the 'carbyne' phase along with different structural forms of sp^2 carbon, mineral impurities etc. The small domain size of the 'carbyne' phase is expected to give rise to broad XRD peaks; this aspect was alleviated to some extent by using a longer wavelength radiation CoK_α (1.789 Å) instead of the standard CuK_α (1.54 Å) and high-resolution optics and beam focusing. Data collection was carried out over a longer period using step-scan with up to 30 seconds per angular step.

The XRD peaks for the carbyne phase were identified using the following criteria (see main text): (a) all relevant peaks should have similar peak shapes and profile clearly distinct from other peaks, and (b) these peaks must only be present in 375°C and 400°C chars and be absent in 600°C chars. Six peaks, marked with the symbol '▲', met these criteria and were used for 'carbyne' structure determination. Peaks located at 24°, 51° and 53.5° (marked with '*') did not belong to the carbyne phase, as these were present at all three temperatures. Diffraction peaks at 31°, 49.5°, 76° and 81° were found to be relatively too sharp.

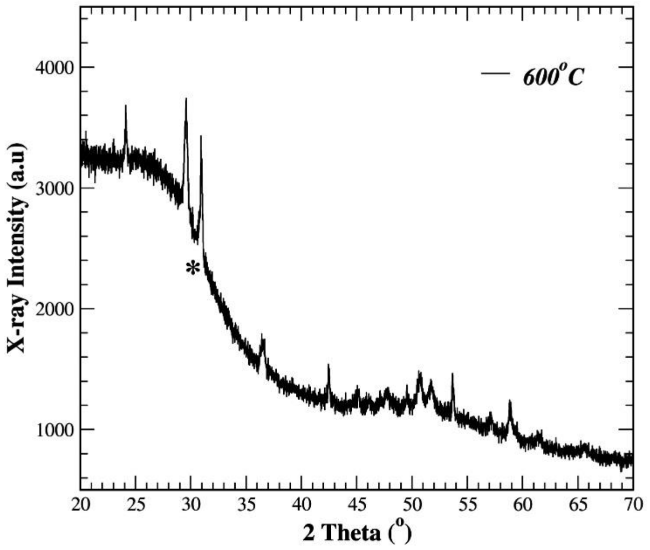

Figure S2a| XRD spectrum for the 600°C lignin char using Co radiation

The XRD spectrum for the 600°C lignin char is shown in Fig. S2a. With the peak at 31° peak (marked with an '*' in the figure) attributed to impurity silica, indexing and structural characterization of other peaks have been detailed in Table S2.

Table S2| Structural characterization of sharp peaks present in 600°C char

Hexagonal: a = 8.83 Å, c = 7.0 Å				
**2 Theta (°)**	**h k l**	**d_o (Å)**	**d_c (Å)**	**d_o-d_c (Å)**
24.49	110	4.394	4.415	-0.021
29.60	002	3.502	3. 5	0.002
36.54	120	2.85	2.89	-0.03
42.43	300	2.47	2.549	-0.079
45.12	003	2.33	2.333	-0.003
51.81	113	2.071	2.063	0.008
53.77	203	1.978	1.99	-0.012
59.0	123	1.817	1.816	0.001

The structural phase of carbon present in 600°C chars was very similar to the phase ‘C’ (a=8.83Å; c=6.9Å) reported previously [21].

Spatial distribution of the ‘Carbyne’ phase

In addition to determining the structure of ‘carbyne’, an attempt was made to determine its spatial distribution as well. After identifying the peaks for the ‘carbyne’ phase, a micro-diffraction investigation was carried out for preparing a spatial map for this phase. Micro XRD measurements were performed on Philips X’pert MRD PRO system with a horizontal high resolution Ω-2θ Goniometer (320 mm radius) with a minimum step size of 0.0001° [2]. A 3-dimensional map of the surface showing the location of phases belonging to the 29.74° carbyne peak in lignin chars is shown in Fig. S2b. The ‘dark brown’ phase in this image represents the location of the carbyne phase in the 400 °C char. The peak intensities were found to be generally quite low. This phase was not found to be localized in any specific region but was found distributed throughout the sample. Similar spatial features were observed in High resolution SEM images as well (Supplementary Section 3).

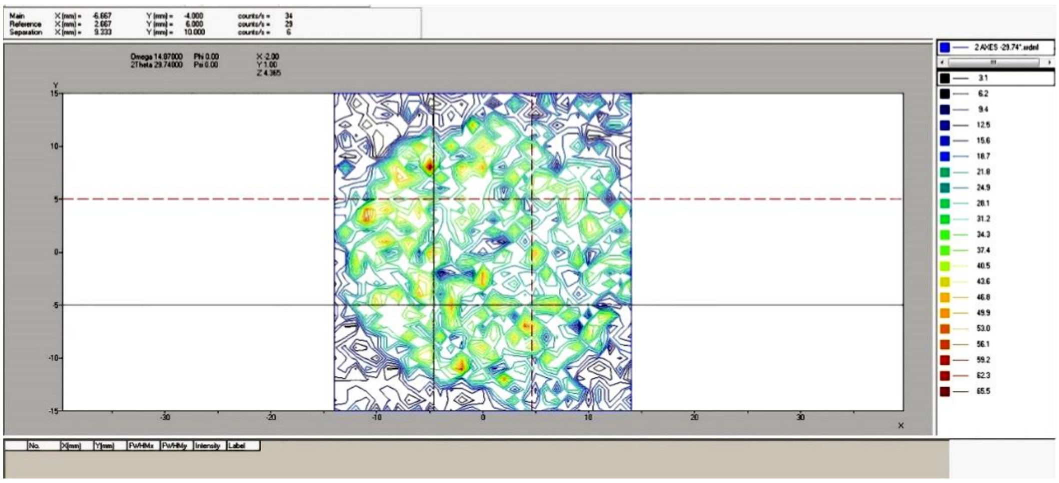

Figure S2b| Epitaxial mapping of 24.79° carbyne peak indicating peak intensities and mapping out local concentrations of the Carbyne phase in 400 °C char.

Supplementary Section S3:

Composition, spatial distribution and the role of impurities

Estimated composition of lignin chars: No attempt was made in this study to estimate the composition of the carbyne phase due to its low concentrations and dispersion in an organic matrix. We focused instead on identifying the key constituents of lignin chars based on the initial composition of raw lignin and estimates for some lignin chars. The ultimate analysis (wt. %) of raw biobased lignin indicated the presence of: C (54.0), N (1.44), S (0.4), H (5.1), O (39.06). A significant proportion of volatiles and moisture will be lost upon heating at 400 °C (weight loss recorded > 40%) [21]. Key constituents present in chars are therefore expected to be C, N, and small amounts of S, H and O. XPS results (at. %) on 400 °C char indicated the presence of 1.7 % N, 11 %O and 0.84% S and balance C. An SEM/EDS analysis of 600°C char is presented in Fig. S3a. Key constituents in this char were found to be predominantly carbon with a very small peak for oxygen.

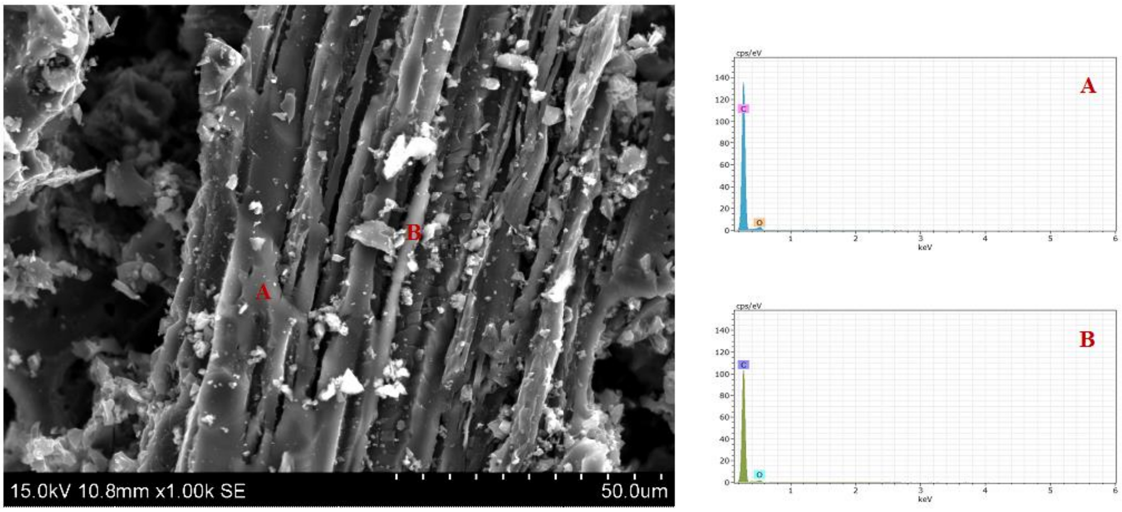

Figure S3a| SEM/EDS images for 600°C chars indicating carbon to be the key constituent, along with a small oxygen peak. White regions in the SEM image indicate ash impurities.

All these results indicate that the key element present in lignin chars was carbon along with a small amount of oxygen, nitrogen, sulphur and mineral impurities. The presence of hydrogen, which cannot be measured accurately during above analysis was indicated in the NMR spectra from all carbon species (Fig. S1a) and proximate analysis.

Spatial distribution of the 'Carbyne' phase: Fig. S3b shows an HRSEM image of the 400°C char showing the spatial distribution of cavities containing the carbyne phase (Fig. 4F). Several such cavities were found distributed across the specimen and were not localized in a specific area. This result on the spatial distribution of the 'carbyne phase' is in good agreement with the epitaxial mapping results from x-ray diffraction (Fig. S2).

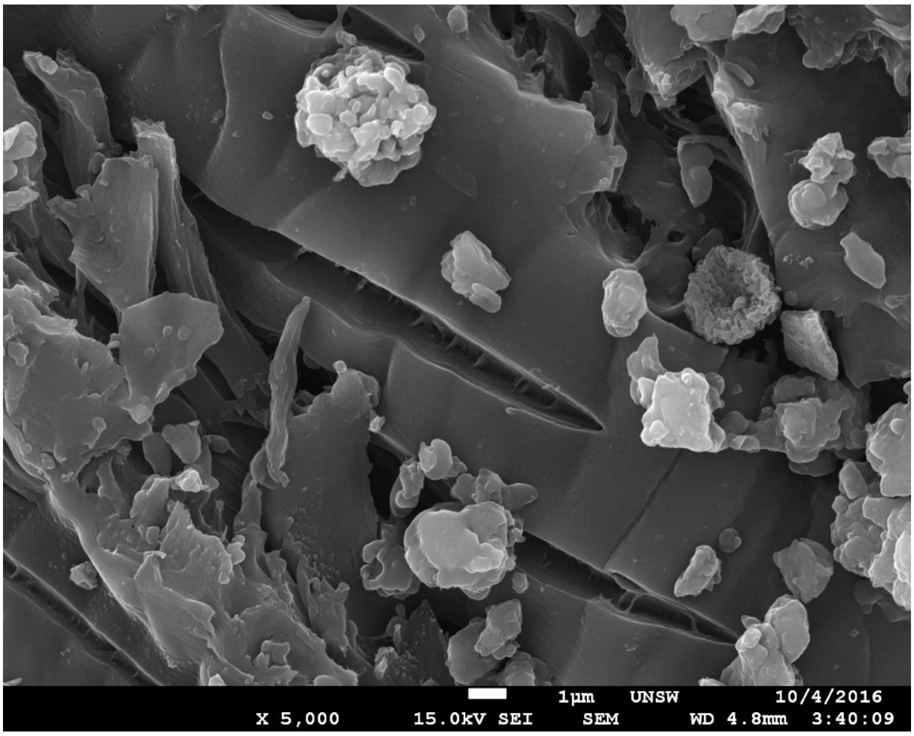

Figure S3b| HRSEM images for 400°C chars indicating the spatial distribution of cavities and the carbyne phase across the specimen.

Role of cellulose and ash impurities: Next, we look at the role, if any, played by the presence of cellulose and ash impurities. Thermal degradation of cellulose has been reported to occur in the temperature range 350 -380 °C.[28] The XRD/SEM signatures from cellulose were not observed in the XRD spectra for 400°C chars. Cellulose is likely to have degraded at 400°C, the thermal stability region for the carbyne phase. However, we cannot comment if cellulose had any influence/role on the nucleation of carbyne phase. This aspect needs to be ascertained with further studies on lignins with or without cellulose. While the whitish ash impurities were clearly identified in SEM micrographs (Fig. S3b), the presence of mineral impurities is expected to have a negligible influence on the formation of linear carbons, probably due to their high thermal stability at low pyrolysis temperatures.

REFERENCES

1. Maciel, G. E. *et al.* A ^{13}C NMR studies of four lignins in the solid and solution states. *Makromol. Chem.* **182**, 2297-2304 (1981).
2. Ikram-Ul-Haq, M. et al. A novel x-ray micro-diffraction approach for structural characterization of trace quantities of secondary phases in Al_2O_3-C/Fe system. *Metallurgical and Materials Transactions B* **45**, 1970-1973 (2014).
